# Supplementary material for: Similarities and differences in patterns of germline mutation between mice and humans
Source: Nat Commun. 2019 Sep 6;10:4053. doi: 10.1038/s41467-019-12023-w (PMC6731245; doi:10.1038/s41467-019-12023-w)
Supplement: Supplementary file 1 — Supplementary Information [file 41467_2019_12023_MOESM1_ESM.pdf]

Similarities and differences in patterns of germline mutation between mice and humans,

Lindsay et al.

Supplementary Data Tables and Figures and Notes

ID	Early Embryonic	peri-PGC	late-postPGC	VEE	total	Corrected total	sex	Age of parents at conception of offspring	Average sequence coverage (WGS)
CBGP4_6a	2	1	8	4	15	14.55	M	37	45
CBGP4_6b	2	2	9	6	19	16.74	M	37	46
CBGP4_6c	3	1	8	2	14	14.39	M	37	42
CBGP4_6d	2	1	13	4	20	19.05	M	37	41
CBGP4_6e	5	2	12	3	22	22.39	F	37	47
CBGP7_2a	3	3	12	3	21	21.12	M	12	38
CBGP7_2b	4	3	10	4	21	20.63	M	12	45
CBGP7_2c	5	2	7	7	21	18.26	M	12	41
CBGP7_2d	5	2	12	6	25	23.69	M	12	39
CBGP7_2e	2	2	6	11	21	17.56	F	12	40
CBGP8_1a	1	3	6	5	15	14.03	M	8	23
CBGP8_1b	0	5	6	7	18	16.47	M	8	35
CBGP8_1c	2	5	8	4	19	18.28	M	8	22
CBGP8_1g	1	6	4	5	16	14.50	F	8	22
CBGP8_1h	1	1	10	2	14	14.17	F	8	23
CBGP8_8a	1	3	13	1	18	19.16	M	40	25
CBGP8_8b	2	3	8	5	18	17.15	M	40	23
CBGP8_8c	0	2	12	5	19	18.01	M	40	23
CBGP8_8d	3	5	19	9	36	33.53	F	40	22
CBGP8_8f	4	5	11	3	23	23.88	F	40	25
GPCB11_1a	1	3	11	3	18	17.77	M	8	41
GPCB11_1b	0	2	7	7	16	13.45	M	8	42
GPCB11_1d	2	3	2	1	8	8.68	M	8	44
GPCB11_1f	4	1	8	5	18	15.80	F	8	42
GPCB11_1g	3	1	15	1	20	21.31	F	8	43
GPCB2_1a	1	0	10	13	24	19.26	M	9	34
GPCB2_1b	1	4	5	4	14	12.75	M	9	32
GPCB2_1c	1	1	15	8	25	23.53	M	9	39
GPCB2_1d	1	9	11	3	24	24.38	M	9	24
GPCB2_1e	1	6	5	1	13	13.88	M	9	40
GPCB2_9a	2	8	12	4	26	24.95	M	41	31
GPCB2_9b	1	2	9	6	18	16.02	M	41	24
GPCB2_9c	0	5	16	7	28	26.86	M	41	22
GPCB2_9e	2	3	22	0	27	29.84	M	41	25
GPCB2_9f	2	3	15	10	30	26.58	F	41	23
GPCB9_8a	0	2	20	11	33	28.03	M	40	40
GPCB9_8c	1	1	12	3	17	16.81	F	40	42
GPCB9_8d	2	0	8	3	13	12.39	F	40	42
GPCB9_8f	2	3	17	5	27	28.40	F	40	40
GPCB9_8g	0	3	11	3	17	16.34	F	40	42

Supplementary Table 1: Mouse DNM counts.

Table showing counts of DNMs in each strata for each individual. Offspring are labelled as a-g in litters *_1 to *_9. *De novo* indels were excluded from the analyses. The column “corrected SNVs” shows the number of autosomal SNVs corrected for the areas of the genome inaccessible to our WGS study, and for likely inheritance of VEEs.

Pedigree	Chr	Pos	Ref	Alt	Mosaic Parent	mean % of reads with mutant allele in parental tissues	Number of offspring with mutation	Number of offspring sampled	lineage	germline mosaic frequency
CBGP4	6	34892512	G	A	Father	5.496	2	41	P11	0.049
CBGP4	12	96555454	T	G	Father	12.668	2	41	P11	0.049
CBGP4	14	97813384	G	A	Father	1.193	1	41	P11	0.024
CBGP4	15	29094924	A	T	Father	9.975	2	41	P11	0.049
CBGP4	18	59431259	G	T	Father	1.644	1	41	P11	0.024
CBGP4	18	63653717	C	T	Father	3.094	1	41	P11	0.024
CBGP4	18	77628350	C	G	Father	0.919	1	41	P11	0.024
CBGP4	2	41430847	C	T	Father	2.342	6	41	P12	0.146
CBGP4	15	12730537	C	A	Father	10.601	9	41	P12	0.22
CBGP4	2	53187077	T	C	Father	10.371	7	41	P13	0.171
CBGP4	12	64770094	T	G	Mother	4.548	7	41	M10	0.171
CBGP4	3	33383613	A	T	Mother	3.366	1	41	M9	0.024
CBGP7	3	43034668	T	G	Father	2.295	2	46	P15	0.043
CBGP7	17	48347925	T	G	Father	2.023	4	46	P15	0.087
CBGP7	18	82381825	A	G	Father	7.455	4	46	P15	0.087
CBGP7	1	109973541	A	T	Father	5.392	9	46	P16	0.196
CBGP7	2	11064217	G	C	Father	5.563	6	46	P16	0.13
CBGP7	5	123117988	C	T	Father	2.528	7	46	P16	0.152
CBGP7	1	3219586	G	A	Father	11.875	14	46	P17	0.304
CBGP7	2	18380082	A	G	Father	12.91	12	46	P17	0.261
CBGP7	17	8698893	G	A	Father	12.678	13	46	P17	0.283
CBGP7	3	70223928	G	A	Father	2.213	1	46	P17	0.022
CBGP7	4	47685275	T	C	Mother	1.19	12	46	M12	0.261
CBGP7	8	118892306	G	T	Mother	1.297	10	46	M12	0.217
CBGP7	9	82120658	C	T	Mother	2.638	11	46	M12	0.239
CBGP8	2	10556273	G	A	Father	2.612	10	57	P1	0.175
CBGP8	2	92472946	A	T	Father	2.122	10	57	P1	0.175
CBGP8	15	60633937	A	C	Father	2.913	7	57	P1	0.123
CBGP8	17	64534541	G	C	Father	2.342	12	57	P1	0.211
CBGP8	4	28191752	T	C	Father	4.05	4	57	P2	0.07
CBGP8	13	61390887	T	C	Father	1.458	2	57	P2	0.035
CBGP8	10	89180326	A	T	Father	9.508	10	57	P3	0.175
CBGP8	17	89395091	G	C	Father	7.695	1	57	P4	0.018
GPCB11	1	138426118	T	C	Father	10.834	10	47	P22	0.213
GPCB11	2	142507389	G	A	Father	10.061	10	47	P22	0.213
GPCB11	2	146721803	G	A	Father	9.818	9	47	P22	0.191
GPCB11	18	32504139	G	A	Father	3.344	6	47	P22	0.128
GPCB11	3	83756086	C	T	Mother	2.684	4	47	M17	0.085
GPCB11	12	53018942	G	T	Mother	1.296	7	47	M17	0.149
GPCB11	8	110980784	G	A	Mother	2.581	4	47	M18	0.085
GPCB11	15	22732768	C	T	Mother	1.926	4	47	M18	0.085
GPCB2	1	195105306	C	T	Father	1.905	8	77	P6	0.104
GPCB2	11	67581517	G	A	Father	9.218	9	77	P6	0.117
GPCB2	12	14764225	C	T	Father	7.395	9	77	P6	0.117
GPCB2	18	68245753	G	T	Father	0.824	13	77	P7	0.169
GPCB2	2	123112871	G	A	Father	4.795	9	77	P8	0.117
GPCB2	11	56513226	A	T	Father	2.512	12	77	P8	0.156
GPCB2	3	27601982	A	C	Father	6.166	6	77	P9	0.078
GPCB2	6	76705056	A	G	Father	0.938	7	77	P9	0.091
GPCB2	4	88898019	T	A	Mother	4.093	10	77	M4	0.13
GPCB9	19	34151974	G	A	Father	10.926	3	77	P19	0.039
GPCB9	15	22224009	A	G	Mother	1.363	1	77	M14	0.013
GPCB9	18	88094408	C	A	Mother	7.681	1	77	M14	0.013
GPCB9	14	14762737	T	G	Mother	3.739	2	77	M15	0.026
GPCB9	14	78127137	A	T	Mother	3.846	2	77	M15	0.026

Supplementary Table 2: Early Embryonic DNMs in mice

Early Embryonic DNMs from six mouse pedigrees, annotated with parent of origin and levels of somatic and germline mosaicism observed. Germline mosaic frequency was calculated as number of offspring carrying mutation/total number of offspring assayed.

chr	pos	ref	alt	ped	Parent of origin	Outcome	Number of Offspring Assayed	Number of Offspring with mutation	Mosaicism	Average variant allele fraction (Offspring)	Variant allele fraction (Parents)
5	40039235	C	G	CBGP4	Father	FAIL	NA	NA	NA	NA	NA
9	9651062	G	T	GPCB11	Father	FAIL	NA	NA	NA	NA	NA
9	51859263	G	A	CBGP7	Mother	FAIL	NA	NA	NA	NA	NA
6	47509685	A	T	GPCB11	Father	FAIL	47	0	0	0	0.07
1	112055045	C	A	CBGP4	Mother	FAIL	40	2	0.05	0.71	0.16
2	58446583	C	T	GPCB9	Father	FAIL	54	18	0.33	0.98	0.28
7	79891839	T	C	GPCB9	Mother	FAIL	55	3	0.05	0.52	0.11
7	95769188	C	A	GPCB9	Mother	FAIL	54	20	0.37	0.5	0.16
12	83079835	A	C	GPCB11	Mother	FAIL	47	14	0.3	0.5	0.06
13	44372937	T	G	GPCB11	Mother	FAIL	47	7	0.15	0.5	0
13	64463785	G	A	CBGP7	Father	FAIL	45	4	0.09	0.49	0.09
17	59986828	C	T	CBGP7	Father	FAIL	46	5	0.11	0.5	0.07
1	140558754	G	A	CBGP7	Father	PASS	46	11	0.24	0.5	0.21
2	38136612	T	A	CBGP7	Father	PASS	46	4	0.09	0.5	0.15
2	75299374	T	A	CBGP4	Father	PASS	40	1	0.03	0.5	0.15
2	89393187	T	A	CBGP7	Mother	PASS	46	13	0.28	0.5	0.13
3	59107159	A	G	GPCB9	Mother	PASS	55	11	0.2	0.5	0.21
4	29438300	T	C	GPCB11	Father	PASS	47	7	0.15	0.5	0.09
4	32934671	C	T	CBGP7	Father	PASS	41	21	0.51	0.5	0.22
4	52046695	G	C	GPCB11	Mother	PASS	47	1	0.02	0.51	0.07
5	146886001	A	T	GPCB9	Mother	PASS	55	9	0.16	0.49	0.19
6	26640451	A	G	CBGP4	Father	PASS	40	5	0.13	0.49	0.24
6	26971358	C	G	GPCB11	Father	PASS	47	4	0.09	0.5	0.23
6	28151992	A	G	GPCB9	Mother	PASS	55	9	0.16	0.44	0.2
6	149428040	T	C	GPCB9	Mother	PASS	55	18	0.33	0.47	0.3
7	119861636	C	T	GPCB9	Mother	PASS	54	16	0.3	0.51	0.31
8	59958418	G	A	GPCB9	Mother	PASS	54	14	0.26	0.5	0.31
8	78366703	G	C	CBGP4	Mother	PASS	40	4	0.1	0.5	0.16
8	87011056	C	T	GPCB11	Mother	PASS	47	0	0	0	0.08
8	127818809	A	G	GPCB9	Mother	PASS	54	7	0.13	0.49	0.21
10	50837657	C	T	CBGP7	Mother	PASS	46	0	0	0	0.15
12	29010841	G	T	CBGP4	Father	PASS	40	7	0.18	0.51	0.08
12	48054417	C	T	GPCB9	Father	PASS	54	20	0.37	0.51	0.28
12	67115109	A	G	CBGP4	Mother	PASS	50	4	0.08	0.48	0.15
14	122391093	T	C	GPCB9	Father	PASS	55	0	0	0	0.09
6	72332353	G	T	GPCB11	Father	PASS	46	0	0	0	0.04
9	80645537	G	A	GPCB9	Mother	PASS	54	0	0	0	0.02
1	88802774	C	G	GPCB9	Father	PASS	52	24	0.46	0.5	0.42
16	15153420	G	T	GPCB9	Father	FAIL	NA	NA	NA	NA	NA
18	51620274	A	G	GPCB9	Mother	FAIL	NA	NA	NA	NA	NA

Supplementary Table 3: Quantification of VEE mutations to the germline.

All 40 putative parental VEE mutations that we attempted to validate are listed. Sites classed as “FAIL” either failed to generate informative data or were not a VEE mutation.

IIDs	chr	position	reference	alternative	consequence	gene	strata
CBGP8_1k, CBGP8_2a, CBGP8_2i, CBGP8_5a, CBGP8_5b, CBGP8_5c, CBGP8_7a, CBGP8_7c, CBGP8_8b, CBGP8_8d	2	10556273	G	A	synonymous SNV	*Sfmbt2*	paternal early embryonic
CBGP8_8f	2	28556769	A	T	nonsynonymous SNV	*Cel*	late post-PGC specification
CBGP8_1a, CBGP8_3a, CBGP8_4a, CBGP8_4h, CBGP8_5d	2	30084760	C	T	nonsynonymous SNV	*Pkn3*	paternal peri-PGC specification
CBGP8_8a	7	104975169	T	C	nonsynonymous SNV	*Olfr671*	late post-PGC specification
CBGP8_1c	8	83722794	G	A	splicing	*Ddx39*	late post-PGC specification
CBGP8_1g	9	123480538	C	T	nonsynonymous SNV	*Limd1*	late post-PGC specification
GPCB2_1d	11	50873775	G	A	stopgain	*Zfp454*	late post-PGC specification
CBGP8_1h	13	100154877	C	T	synonymous SNV	*Naip2*	late post-PGC specification
CBGP8_1h	13	100154880	G	A	synonymous SNV	*Naip2*	late post-PGC specification
CBGP8_1h	13	100154911	T	A	nonsynonymous SNV	*Naip2*	late post-PGC specification
CBGP8_1h	13	100154951	C	T	nonsynonymous SNV	*Naip2*	late post-PGC specification
CBGP8_1a	19	44935143	G	A	nonsynonymous SNV	*Fam178a*	late post-PGC specification
CBGP7_2e	2	76889786	T	C	nonsynonymous SNV	*Ttn*	VEE
CBGP4_6e	3	146944022	G	A	synonymous SNV	*Ttll7*	VEE
CBGP4_6c, CBGP4_6e	6	34892512	G	A	nonsynonymous SNV	*Wdr91*	paternal early embryonic
GPCB9_8g	7	12417167	T	C	nonsynonymous SNV	*Zfp551*	late post-PGC specification
GPCB11_1d, GPCB11_4c	7	27465841	C	T	synonymous SNV	*Blvrb*	peri-PGC specification
GPCB9_8f	9	106224354	T	A	nonsynonymous SNV	*Tlr9*	late post-PGC specification
GPCB9_8c	10	5075830	C	T	nonsynonymous SNV	*Syne1*	late post-PGC specification
CBGP7_2a, CBGP7_2b, CBGP7_6b, CBGP7_8e	10	81261530	C	T	nonsynonymous SNV	*Matk*	peri-PGC specification
CBGP7_2a, CBGP7_2b	11	115802091	G	A	nonsynonymous SNV	*Caskin2*	peri-PGC specification
CBGP7_2a	13	105250190	C	T	synonymous SNV	*Rnf180*	late post-PGC specification
CBGP4_6a	17	24600010	A	C	nonsynonymous SNV	*Tsc2*	late post-PGC specification
CBGP7_2e	17	25492005	G	A	synonymous SNV	*Sstr5*	VEE
CBGP7_2e	18	64561658	A	C	nonsynonymous SNV	*Atp8b1*	late post-PGC specification
CBGP7_2b	19	7281156	G	T	synonymous SNV	*Mark2*	late post-PGC specification

Supplementary Table 4: DNMs with potentially functional consequences as given by ANNOVAR[29] are listed.

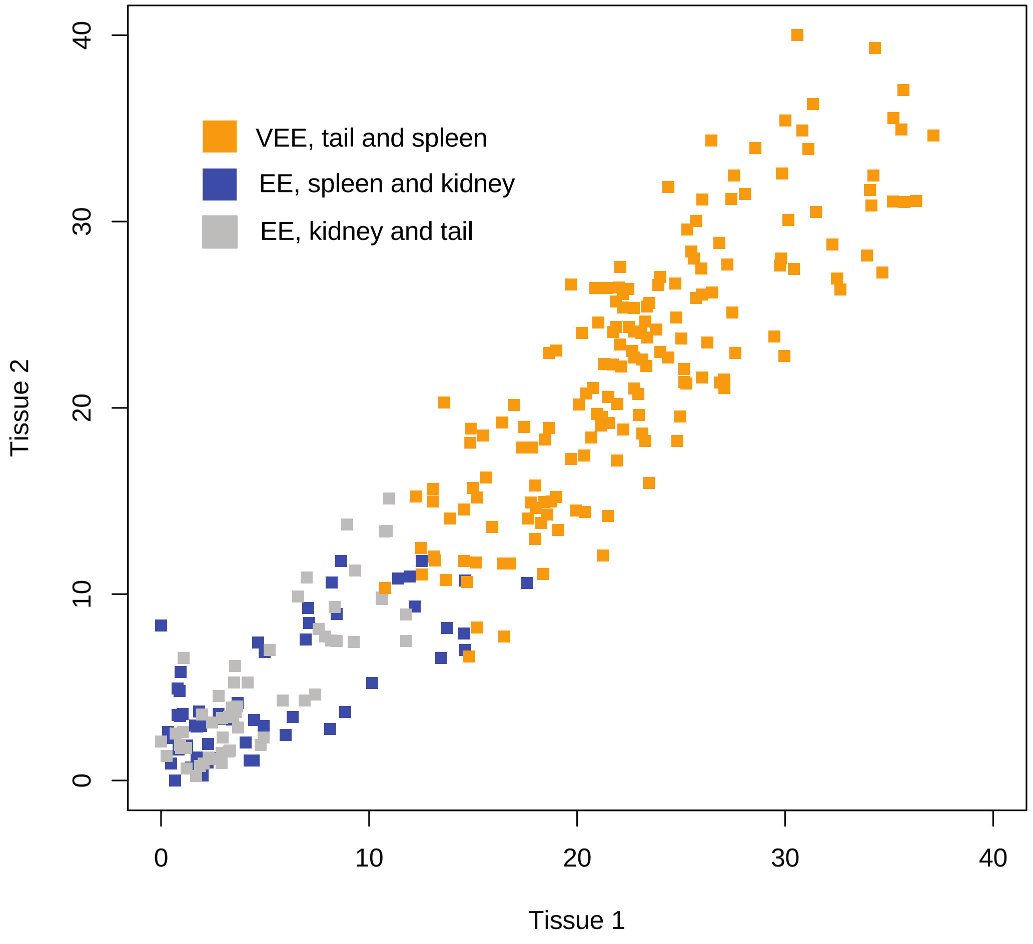

Supplementary Figure 1. Consistency of VAF across 2 tissues. The percentage of reads with the alternative allele in tissue 1 is plotted against the percentage of reads with the alternative allele in tissue 2. For VEE mutations, the mutation was typed in two tissues (spleen and tail) in the offspring. EE mutations were typed in spleen, tail and kidney in the parental tissues.

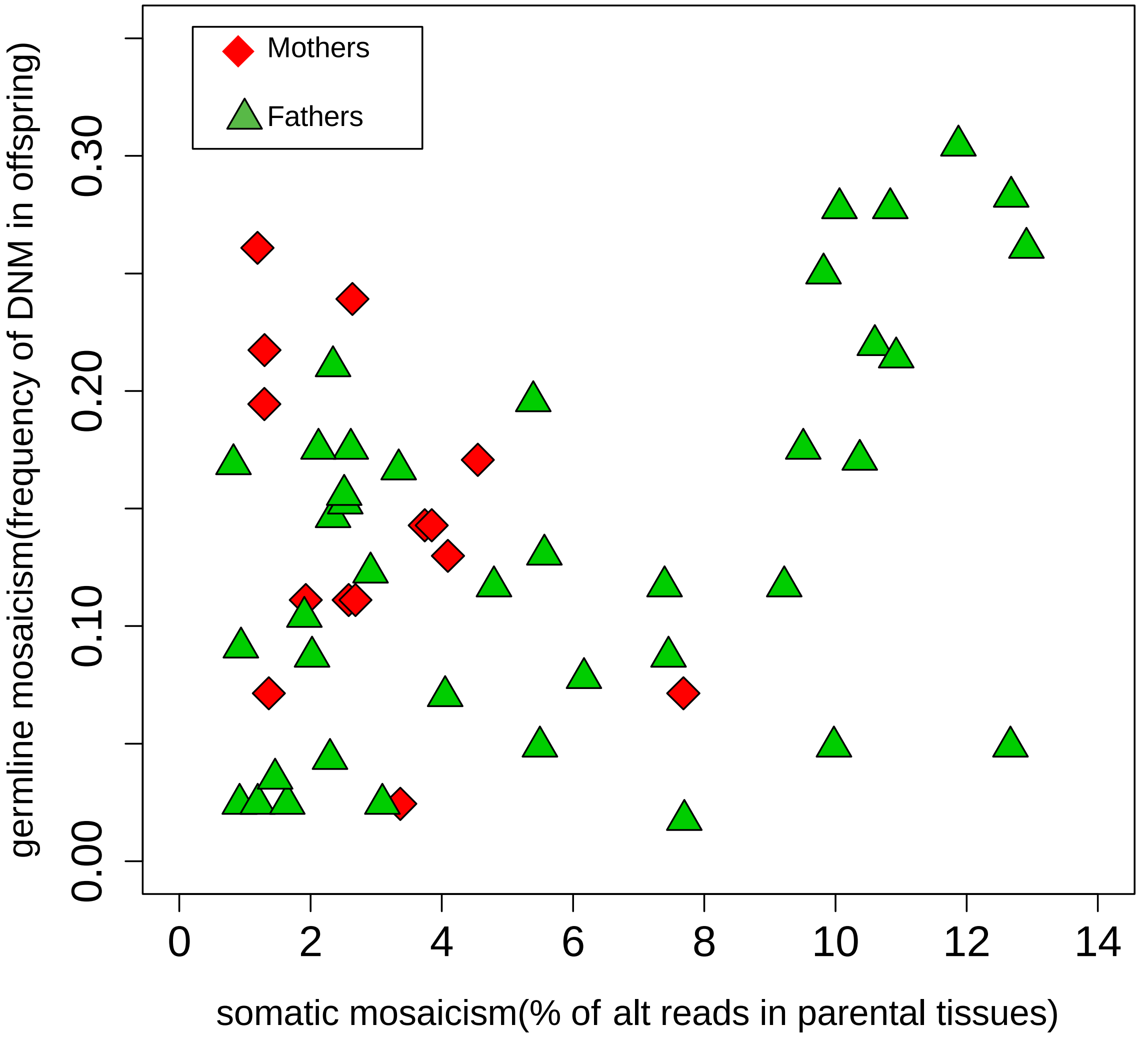

Supplementary Figure 2: **Somatic and Germline mosaicism observed at 55 EE mutations in six mouse pedigrees.**

The y axis shows the germline mosaicism (number of DNMs in offspring/total number of offspring genotyped). The x axis shows the % of alternate reads observed in parental tissues.

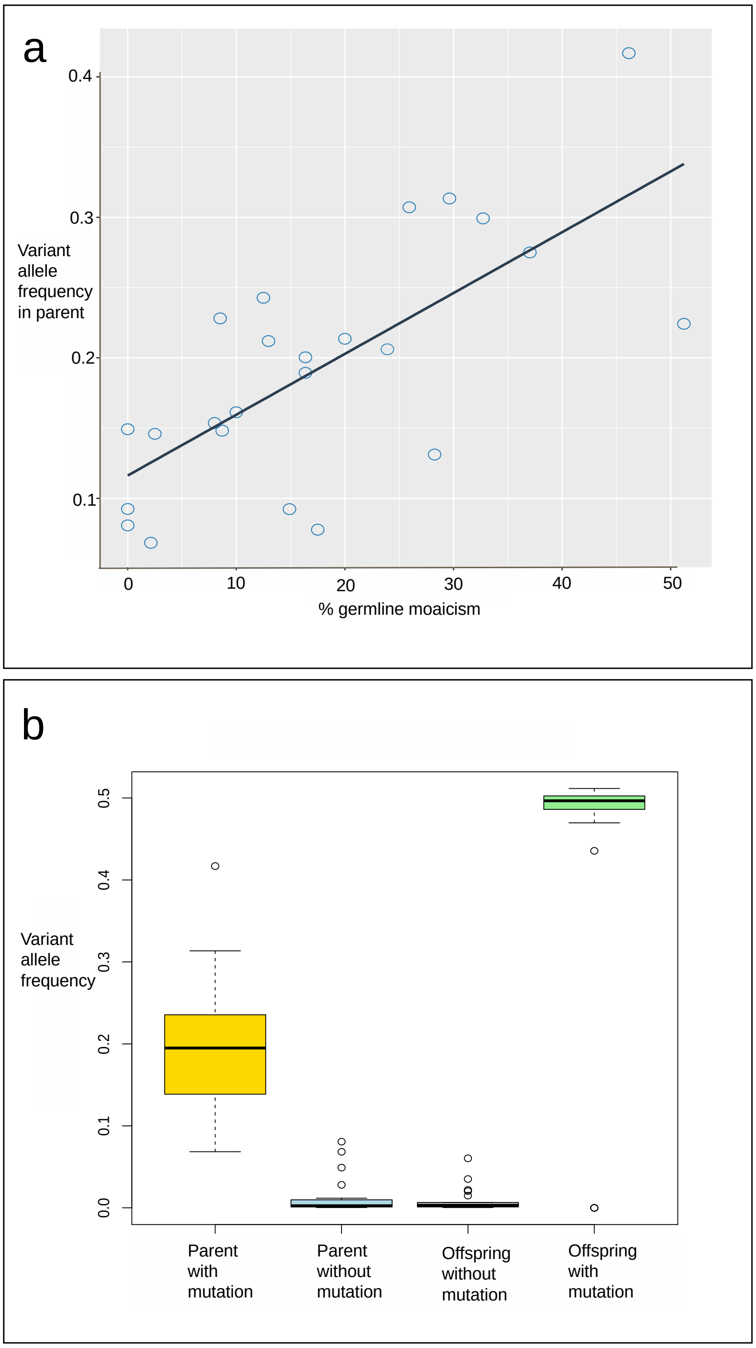

Supplementary Figure 3: Germline mosaicism of VEE mutations observed in the parents. (a) The frequency of the variant allele in the parent is plotted against the % of offspring assayed carrying the variant allele as an inherited constitutive variant. **(b)** Boxplots of the frequency of the variant allele in the parents and offspring, demonstrating the distinction between the variant allele frequency observed as embryonic mutations in the parents and as constitutive variants in the offspring. The midline shows the median, while is delineated by the first and third quartiles, the whiskers 1.5X the interquartile distance while individual points show outliers.

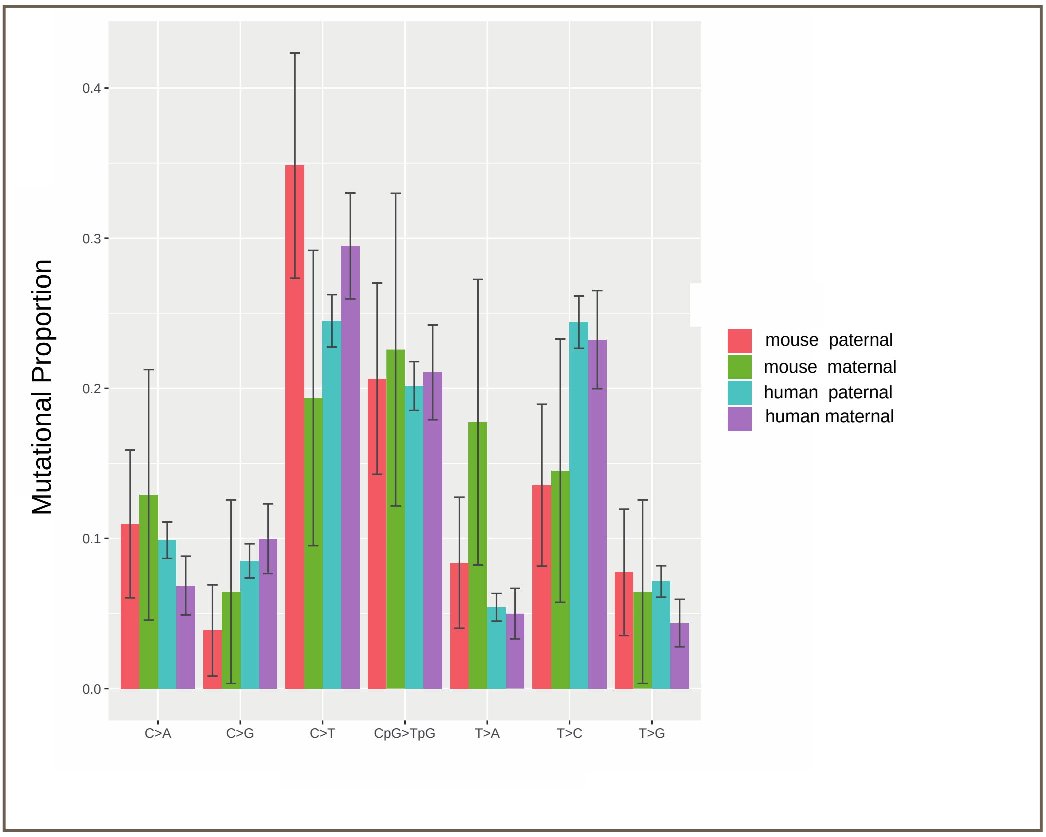

Supplementary Figure 4: Low resolution mutation spectra in maternal and paternally derived DNMs in mouse and human[3] data. Error bars show the 95% confidence intervals.

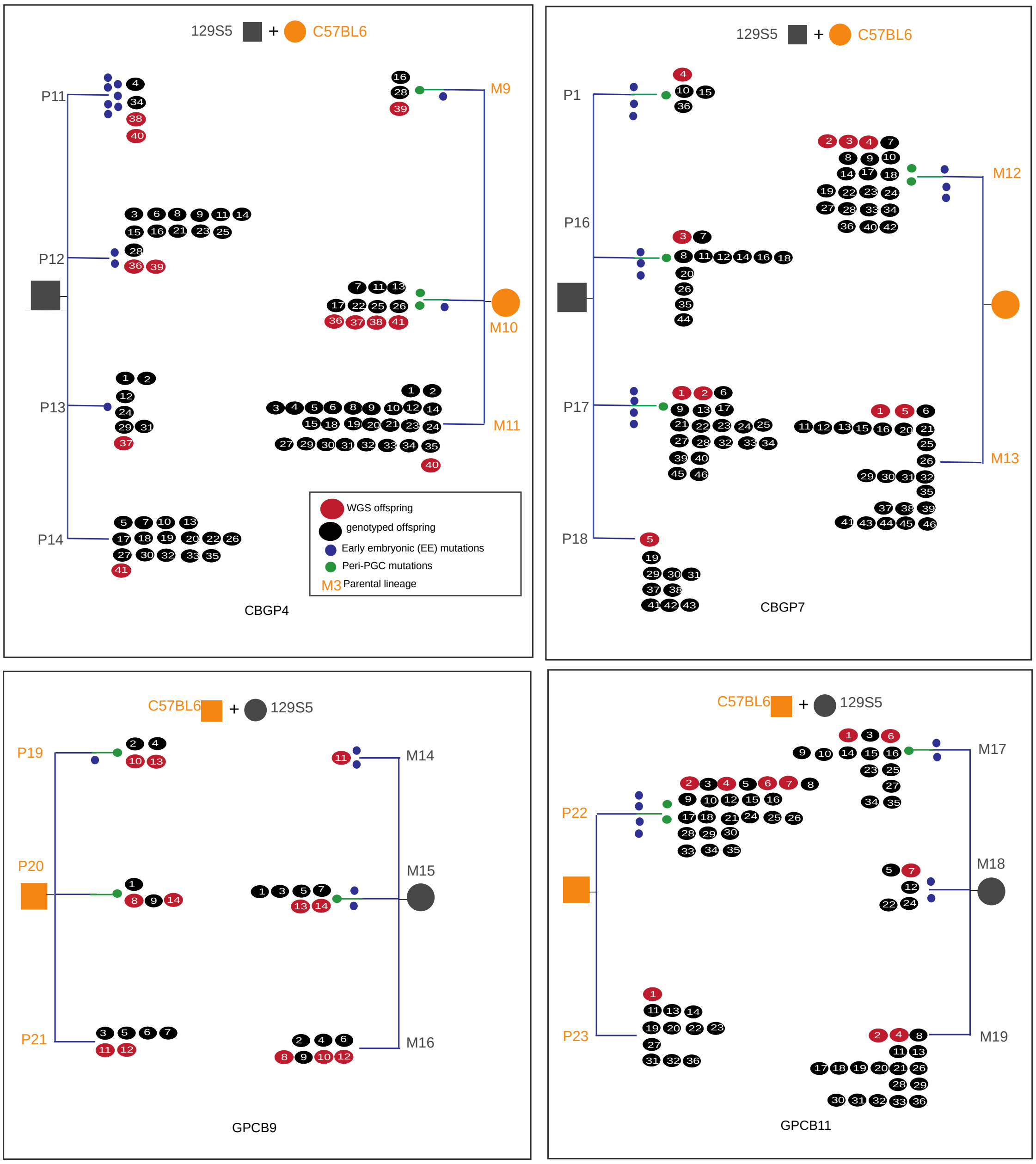

Supplementary Figure 5: Genealogies of maternal and paternal cell lineages delineated by early embryonic and peri-PGC mutations.

Parental embryonic lineages reconstructed for each pedigree as paternal (P) or maternal (M), with each lineage numbered. Mutations delineating the lineages are colour-coded according to their temporal strata are listed in Extended Data Table 5. WGS and genotyped offspring are shown as red and black numbered circles respectively. Offspring are numbered and ordered according to litter; for example. Lineages P14, M11, P18, M13, P21, M16, P23 and M19 represent offspring without shared mutations and may contain several uncharacterised lineages.

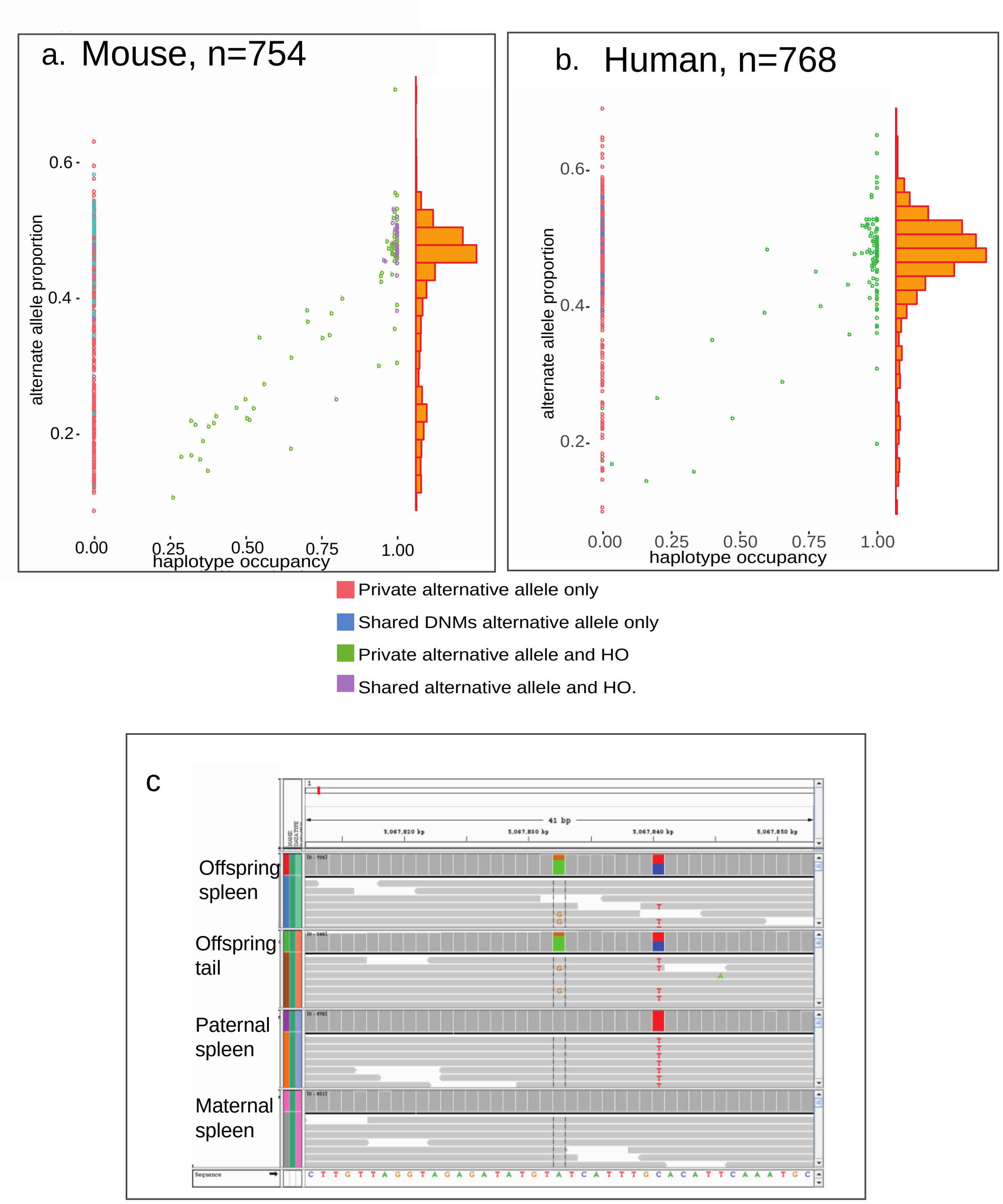

Supplementary Figure 6. Haplotype Occupancy.

(a) Haplotype occupancy of DNMs in mice (haplotypes defined by adjacent heterozygous variants, Methods) plotted against the Variant Allele Fraction of the validated DNM. The right-hand histogram shows the distribution of VAF for DNMs for which haplotype occupancy could be determined. **(b)** Haplotype occupancy of DNMs in humans, **(c)** An example of incomplete haplotype occupancy defined as a DNM (in this case, A->G), which is not present on every instance of the haplotype (defined by the C->T variant) on which it arose.

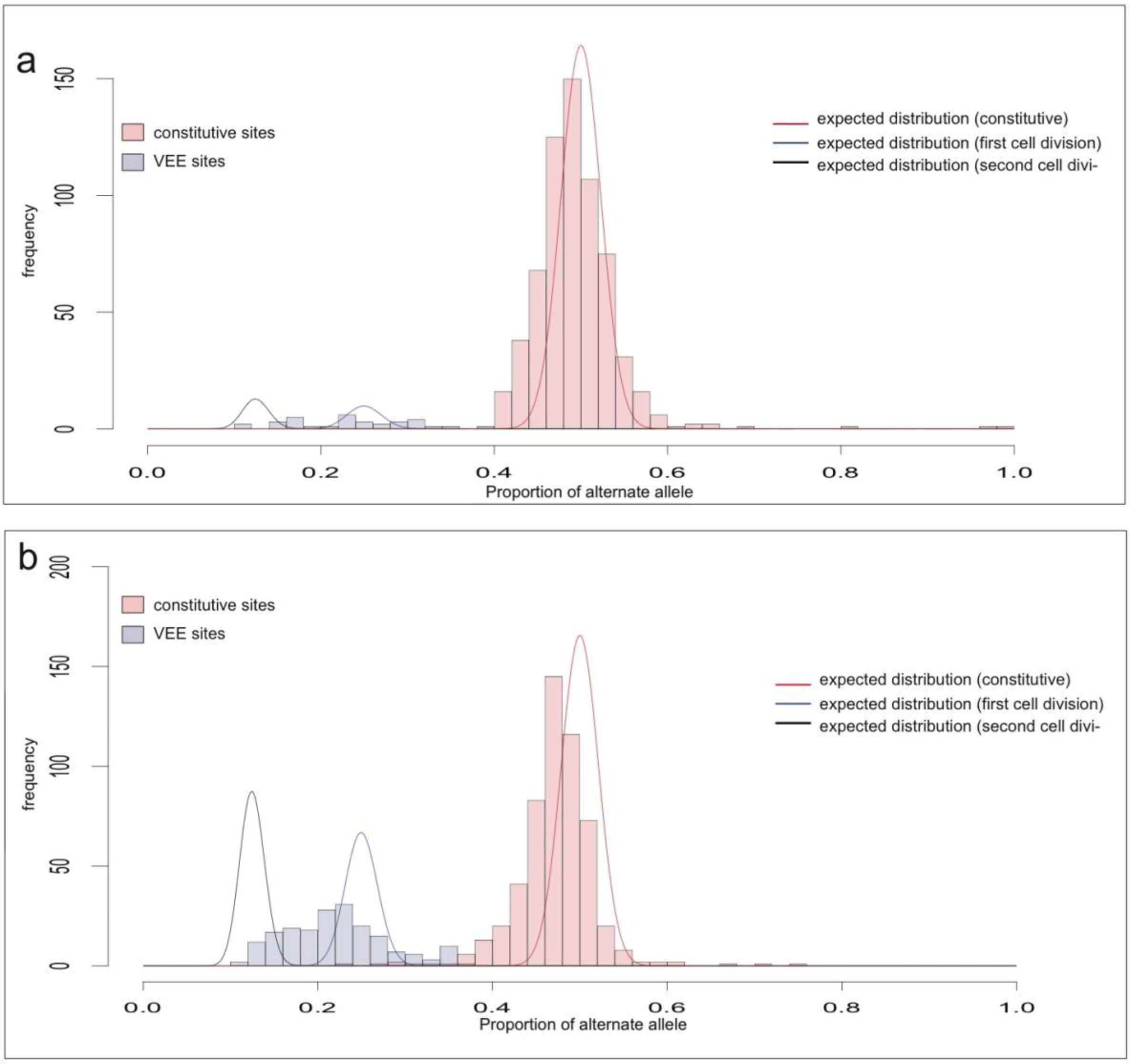

Supplementary Figure 7: Variant Allele Fractions in Deep Sequenced Data. Histograms of the variant allele fraction in validated DNMs in the deep sequence data in humans (A) and mice (B). Constitutive sites are in red and have a VAF centred around 50% of reads (100% of cells). Very early embryonic (VEE) are shown in blue, are found in around 25% of reads (50% of cells). Red, blue and black lines show the expected distribution of VAF given a binomial distribution of reads centred around constitutive, first division and second division mutations (assuming symmetric contributions to the embryo), in our validation sequence data for all pedigrees.

Supplementary Note 1.

We validated 366 unique *de novo* mutations in the CBGP4, CBGP7, GPCB9 and GPCB11 including 4 MNVs and 362 SNVs. We found 38/366 (13%) unique DNMs were EEs in the four pedigrees ranging from 1-13% of parental somatic cells. We detected 31/366 (8%) unique DNMs as peri-PGC (Paternal: Maternal 7:7 read pair phasing). Lastly, we observed 205/386 observed DNMs as late-post PGC mutations. 92/386 observed DNMs (23%) were VEEs, also showed high variance of 1-11 DNMs per individual, more than expected under a Poisson distribution (p=0.037), all pedigrees combined (p=0.0019). They arose at the same rates in male and female offspring 4:4 and at similar rates on parental haplotypes 3:4 (Maternal: Paternal).
