# Supplementary material for: Similarities and differences in patterns of germline mutation between mice and humans
Source: Nat Commun. 2019 Sep 6;10:4053. doi: 10.1038/s41467-019-12023-w (PMC6731245; doi:10.1038/s41467-019-12023-w)
Supplement: Supplementary file 3 — Reporting Summary [file 41467_2019_12023_MOESM3_ESM.pdf]

nature research

Corresponding author(s): Sarah Lindsay

Last updated by author(s): 2019/07/19

Reporting Summary

Nature Research wishes to improve the reproducibility of the work that we publish. This form provides structure for consistency and transparency in reporting. For further information on Nature Research policies, see Authors & Referees and the Editorial Policy Checklist.

Statistics

For all statistical analyses, confirm that the following items are present in the figure legend, table legend, main text, or Methods section.

n/a	Confirmed	
☐	☒	The exact sample size (*n*) for each experimental group/condition, given as a discrete number and unit of measurement
☒	☐	A statement on whether measurements were taken from distinct samples or whether the same sample was measured repeatedly
☐	☒	The statistical test(s) used AND whether they are one- or two-sided<br>*Only common tests should be described solely by name; describe more complex techniques in the Methods section.*
☐	☒	A description of all covariates tested
☐	☒	A description of any assumptions or corrections, such as tests of normality and adjustment for multiple comparisons
☐	☒	A full description of the statistical parameters including central tendency (e.g. means) or other basic estimates (e.g. regression coefficient) AND variation (e.g. standard deviation) or associated estimates of uncertainty (e.g. confidence intervals)
☐	☒	For null hypothesis testing, the test statistic (e.g. *F*, *t*, *r*) with confidence intervals, effect sizes, degrees of freedom and *P* value noted<br>*Give P values as exact values whenever suitable.*
☒	☐	For Bayesian analysis, information on the choice of priors and Markov chain Monte Carlo settings
☒	☐	For hierarchical and complex designs, identification of the appropriate level for tests and full reporting of outcomes
☒	☐	Estimates of effect sizes (e.g. Cohen's *d*, Pearson's *r*), indicating how they were calculated

Our web collection on statistics for biologists contains articles on many of the points above.

Software and code

Policy information about availability of computer code

Data collection	SAMtools, DeNovoGear, Platypus
Data analysis	Intergrative Genomics Viewer (IGV), Annovar, Bedtools,Python, R

For manuscripts utilizing custom algorithms or software that are central to the research but not yet described in published literature, software must be made available to editors/reviewers. We strongly encourage code deposition in a community repository (e.g. GitHub). See the Nature Research guidelines for submitting code & software for further information.

Data

Policy information about availability of data

All manuscripts must include a data availability statement. This statement should provide the following information, where applicable:

- Accession codes, unique identifiers, or web links for publicly available datasets
- A list of figures that have associated raw data
- A description of any restrictions on data availability

The whole genome sequences generated as part of this study were deposited in the European Nucleotide Archive (ENA), under study PRJEB1407 and PRJEB14877.

Field-specific reporting

Please select the one below that is the best fit for your research. If you are not sure, read the appropriate sections before making your selection.

☒ Life sciences ☐ Behavioural & social sciences ☐ Ecological, evolutionary & environmental sciences

For a reference copy of the document with all sections, see nature.com/documents/nr-reporting-summary-flat.pdf

Life sciences study design

All studies must disclose on these points even when the disclosure is negative.

Sample size	Analysis of three multi-sibling human families was already published, so the rationale was that a comparable number of mouse pedigrees would be sufficient.
Data exclusions	No data was excluded from the study
Replication	We replicated the most significant findings of our initial study on two mouse pedigrees by extended the work to include a four further mouse pedigrees.
Randomization	Randomization is not relevant to this observational study.
Blinding	No blinding was carried out in this observational study.

Reporting for specific materials, systems and methods

We require information from authors about some types of materials, experimental systems and methods used in many studies. Here, indicate whether each material, system or method listed is relevant to your study. If you are not sure if a list item applies to your research, read the appropriate section before selecting a response.

Materials & experimental systems

n/a	Involved in the study	
☒	☐	Antibodies
☒	☐	Eukaryotic cell lines
☒	☐	Palaeontology
☐	☒	Animals and other organisms
☐	☒	Human research participants
☒	☐	Clinical data

Methods

n/a	Involved in the study	
☒	☐	ChIP-seq
☒	☐	Flow cytometry
☒	☐	MRI-based neuroimaging

Animals and other organisms

Policy information about studies involving animals; ARRIVE guidelines recommended for reporting animal research

Laboratory animals	This study used wild type mice from two inbred strains CB57BL/6 and 129S5, the parents were allowed to breed over the course of their natural lifespan. Offspring were culled at six weeks.
Wild animals	No wild animals were used in this study.
Field-collected samples	No field collected samples were used in this study.
Ethics oversight	Ethical guidance and approval from Institute ethical boards AWERB and HMDMC

Note that full information on the approval of the study protocol must also be provided in the manuscript.

Human research participants

Policy information about studies involving human research participants

Population characteristics	Three generation families with multiple siblings in the middle generation and ideally with each sib having at least one offspring (grandchildren). Only data required: Pedigree (including sex and relative age within each family)
Recruitment	See above, families were recruited to maximize number of siblings with no other characteristics required.
Ethics oversight	HMDMC, covered under the GS:SFHS Research Tissue Bank 10/S1402/20.

Note that full information on the approval of the study protocol must also be provided in the manuscript.
