# Supplementary material for: Similarities and differences in patterns of germline mutation between mice and humans
Source: Nat Commun. 2019 Sep 6;10:4053. doi: 10.1038/s41467-019-12023-w (PMC6731245; doi:10.1038/s41467-019-12023-w)
Supplement: Supplementary file 4 — Description of Additional Supplementary Files [file 41467_2019_12023_MOESM4_ESM.docx]

**Description of Additional Supplementary Files**

**Supplementary Data 1**: Validated DNMs observed in six mouse pedigrees. All DNMs are listed, with columns in order of chromosome, position, type, reference allele, variant allele, which offspring they were called in (the suffix ‘T’ on the offspring ID, e.g. CBGP8_1aT, indicates that tail DNA rather than spleen DNA was assayed), the number of individuals the site is shared with, the temporal strata to which it was assigned, which lineage it belongs to, and finally read-pair haplotyping results. CBGP7_2a has information from only one tissue (tail) due to a QC problem with DNA from the spleen)
